# The differential placental expression of *ERp44* and pre-eclampsia; association with placental zinc, the *ERAP1* and the renin-angiotensin-system

**DOI:** 10.1016/j.placenta.2023.02.006

**Published:** 2023-03-24

**Authors:** Rhea Raghu, Lesia O. Kurlak, Eun D. Lee, Hiten D. Mistry

**Affiliations:** a Tenafly High School, Tenafly, USA; b Stroke Trials Unit (School of Medicine), University of Nottingham, Nottingham, UK; c Virginia Commonwealth University School of Medicine, Richmond, USA; d Division of Women and Children's Health, School of Life Course and Population Sciences, King's College London, London, UK

**Keywords:** Hypertension in pregnancy, Placenta, *ERp44*, Endoplasmic reticulum, Micronutrient, Angiotensin

## Abstract

**Introduction:**

Endoplasmic reticulum resident protein 44 (ERp44) is a zinc-metalloprotein, regulating Endoplasmic reticulum aminopeptidase 1 (ERAP1) and Angiotensin II (Ang II). We explored placental *ERp44* expression and components of the renin-angiotensin-system (RAS) in pre-eclampsia (PE), correlating these to *ERAP1* expression and placental zinc concentrations.

**Methods:**

Placental tissue, taken at time of delivery in normotensive women or women with PE (n = 12/group), were analysed for *ERp44, AT1R, AT2R* and *AT4R* by qPCR. Protein *ERp44* expression was measured by immunohistochemistry and compared to previously measured *ERAP1* expression. Placental zinc was measured by inductively-coupled-mass-spectrometry.

**Results:**

*ERp44* gene/protein expression were increased in PE (P < 0.05). *AT1R* expression was increased (P = 0.02) but *AT4R* decreased (P = 0.01) in PE, compared to normotensive controls. A positive association between *ERp44* and *AT2R* expression was observed in all groups. *ERp44* was negatively correlated with *ERAP1* protein expression in all samples. Placental zinc concentrations were lower in women with PE (P = 0.001) and negatively associated with *ERp44* gene expression.

**Discussion:**

Increased placental ERp44 could further reduce ERAP1 release in PE, potentially preventing release of Ang IV and thus lowering levels of Ang IV which consequently diminishes the possibility of counterbalancing the activity of vasoconstrictive, Ang II. The lower placental zinc may contribute to dysfunction of the *ERp44*/*ERAP1* complex, exacerbating the hypertension in PE.

## Highlights

• Placental ERp44, zinc, ERAP1, and renin-angiotensin-system in pre-eclampsia.
• ERp44 gene/protein and AT1R expression increased in women with pre-eclampsia.
• AT4R and placental zinc concentrations decreased compared to normotensive controls.
• Suggests possibility for monitoring zinc intakes in women prone to pre-eclampsia.
• Zinc may regulate ERp44, ERAP1 and renin-angiotensin system functions in pregnancy.

## 1 Introduction

Pre-eclampsia (PE) is a hypertensive disorder of pregnancy that occurs in 2–4% of all pregnancies; it is associated with the greatest risk of maternal and fetal morbidity and mortality of all pregnancy-related hypertensive disorders [1]. PE presents with new-onset hypertension and proteinuria in the mother after the 20th week of gestation. It can progress to multi-organ dysfunction, including hepatic, renal and cerebral disease, if the fetus and placenta are not delivered [2]. The hallmark of PE is maternal endothelial dysfunction due to circulating factors of fetal origin from the placenta [3]. PE has lifelong consequences for both the mother and her child, such as increased risks of cardiovascular, renal, and metabolic health problems [4].

*ERp44* is a chaperone of the protein disulfide isomerase (PDI) family and an endoplasmic reticulum thioredoxin (TRX)-like motif-containing protein. It plays key roles in thiol-mediated retention and maturation of several secretory proteins within the endoplasmic reticulum (ER) [5]. *ERp44* cycles between the ER and *cis-*Golgi compartments to patrol proteins and control the traffic and oligomeric assembly of various secretory proteins, including IgM and adiponectin [6].

*ERAP1* is expressed ubiquitously in all tissues including placenta [7]. The enzyme plays an essential role in multiple biological processes which require cleavage of N-terminal amino acid residues. Such processes include the regulation of blood pressure, angiogenesis, the shedding of cytokine receptors, and immune recognition.

*ERp44* also contributes to the control of blood pressure by regulating circulating Angiotensin II (Ang II) levels, which acts differentially through type 1 (AT1R) and type 2 (AT2R) receptors to regulate blood pressure and fluid homeostasis via the renin-angiotensin system (RAS). The balance between the vasoconstrictive and vasodilatory arms of the RAS is disrupted in PE [8,9].

ER aminopeptidase 1 (*ERAP1*) cleaves Ang II to Ang III and the vasodilatory Ang IV, the latter acting through its Angiotensin type 4 receptor (AT4R) [10]. We have previously reported lower expression of *ERAP1* placental protein expression in women PE [7], as well as alterations in the placental RAS in PE [8,11,12]. *ERp44* also complexes with several ER-resident enzymes that lack an ER-retention motif, such as *ERAP1*, with *ERp44* controlling the release of *ERAP1* in a redox-dependent manner [5,13]. This *ERp44*-*ERAP1* interaction has been shown to regulate blood pressure, as *ERp44* suppresses the release of *ERAP1*. Thus, dysregulation of *ERp44* leading to *ERAP1* suppression will act downstream to prevent the cleavage of Ang II, contributing to RAS-induced hypertension [5].

Zinc and zinc ions (Zn^2+^) are crucial elements of metalloenzymes and vital for successful embryogenesis and essential antioxidant activity [14,15]. Zinc is retained during pregnancy with estimated concentrations of around 100 mg total [16], with 57% accrued in the fetus, 6.5% in the placenta, <1% in the amniotic fluid, 24% in the uterus, 5% in mammary tissue, and 6.5% in the expanded maternal blood volume [17]. Zinc concentrations are increased approximately 2-fold during the third trimester when compared to non-pregnant women [18]. Zinc deficiency has been associated with PE [14,15], with some data suggesting zinc level could be a useful clinical marker for severity of PE [19]. The zinc metal ion also serves as a cofactor for stabilizing the three-dimensional structures of proteins [[20], [21], [22]]. including *ERp44*. Zn^2+^ binds *ERp44* with sub-micromolar affinity, regulating its traffic and activity [13]. Alterations in Zn^2+^ have been shown to disrupt *ERp44* function and its complex with *ERAP1* [13], but this relationship has not yet been investigated in the placenta or in relation to PE. Thus, we hypothesize that in PE the decreased placental expression of *ERAP1* will be due to increased *ERp44* expression induced by zinc dysregulation. This increased *ERp44* expression will prevent the conversion of Ang II to its vasodilatory metabolite products, contributing to RAS hyperactivity and maternal hypertension. Furthermore, due to the known association with the RAS, we expect to observe a correlation between *ERp44* expression and Ang II receptor expression, further exacerbating blood pressure dysregulation. Hence, in this study we firstly investigated the mRNA abundance and protein expression of *ERp44* in normotensive pregnant women compared to those with PE: secondly, we explored the relationship between this expression to components of the RAS, as well as to *ERAP1* and placental zinc concentrations.

## 2 Materials and methods

### 2.1 Participants

This study was carried out under the HRA-REC approval gained by the University of Nottingham (REF: 15/EM/0523). All methods were carried out in accordance with relevant guidelines and regulations. Fully informed, signed consent was obtained from all participating women. PE (n = 12) was defined as systolic blood pressure ≥140 mm Hg and diastolic blood pressure ≥90 mmHg, determined on 2 occasions >4 h apart and arising after 20 weeks of gestation in a previously normotensive woman and *de novo* proteinuria (protein: creatinine ratio (PCR) > 30; urine protein concentration >3 g/L in 2 random clean-catch midstream specimens collected >4 h apart) with no evidence of urinary tract infection [23]. No women had any underlying renal or hypertensive disease before 20 weeks gestation. Healthy normotensive pregnant women matched for age (n = 12) who had no prior pregnancy complications, and no evidence of any urinary tract infections were recruited as comparative controls.

Medical and obstetric histories, including delivery data, were obtained from each woman. A summary of the demographic and pregnancy outcome of the women recruited in this study are presented in Table 1. Full-depth placental tissue biopsies were collected within 10 min of the placenta being delivered. Samples were collected from a standardized location midway between the cord insertion and placental border and were either snap frozen for gene expression or processed for immunohistochemistry as previously described [24].

**Table 1:** Demographic, clinical and biochemical data of participants.

Parameter	Normotensive (n = 12)	Pre-eclampsia (n = 12)
Maternal age (years)	32 ± 6.1	30 ± 7.9
Booking body mass index (kg/m^2^)	26.5 ± 4.8	29.7 ± 6.8
Max. systolic blood pressure outside labor (mm Hg)	129 ± 6.1	153 ± 9.2 **
Max. diastolic blood pressure outside labor (mmHg)	83 ± 6.4	99 ± 5.2 **
Protein: creatinine ratio (g/mmol)	–	192 [94, 262]
Gestation age at delivery (weeks)	39.2 ± 0.5	36.4 ± 2.7 *
Caesarean section (No. (%))	8 (67)	10 (83)
Number of male babies (No. (%))	4 (33)	8 (67)
Birthweight (kg)	3.65 [3.37, 3.94]	2.55 [2.01, 3.06] *

*P < 0.05; **P < 0.0001 between normotensive and pre-eclampsia diagnostic groups. Samples matched with previously published *ERAP1* data [7].

### 2.2 RNA extraction, cDNA synthesis and quantitative real time polymerase chain reaction (qPCR)

Total RNA was extracted from ∼100 mg placental tissue using QiAzol lysis reagent (Qiagen, UK) as previously described [25]. RNA (1 μg) was reverse transcribed using the QuantiTect Reverse Transcription kit (Qiagen, UK) in a Primus96 thermocycler (Peqlab Ltd, UK). Real-time PCR was carried out using SYBR Green chemistry (2x QuantiFast SYBR Green, Qiagen, UK) on a AB7500 Fast (Life Technologies, UK) using primers to *ERp44*, *AT1R*, *AT2R* and *AT4R* (Table 1).

*ERAP1* was previously measured in the same samples, using identical methodology [7]. Abundance data for the genes of interest were expressed as normalized copy number following normalization using GeNORM (http://medgen.ugent.be/∼jvdesomp/genorm/), with stably expressed reference genes [26] beta-2 microglobulin (B2M), Tyrosine 3-Monooxygenase/Tryptophan 5-Monooxygenase Activation Protein Zeta (YWHAZ) and Glyceraldehyde 3-phosphate dehydrogenase (GADPH) (Supplementary Table 1).

### 2.3 Immunohistochemical staining

Placental protein expression was assessed by immunohistochemistry as previously described [25], using antibody to *ERp44* (Sigma-Prestige, rabbit monoclonal: HPA001318; 30 μg/ml). As with gene expression, protein expression of *ERAP1* was previously measured in the same samples [7]. All slides were assessed by the same observer, blinded to the participant groups. Quantification was performed as described previously [25,27], using the Positive Pixel Algorithm of Aperio ImageScope software; a visual check was also performed.

### 2.4 Placental zinc concentrations

Placental tissue concentrations of Zn^2+^ (μg/kg of dry matter (DM) were also measured by ICP-MS (intra-assay variability <2%), after prior digestion of ∼400 mg of freeze-dried tissue, to determine the percentage of water content, and subsequent digestion using 2% nitric acid as previously described [28,29]. Certified reference material (NIST SRM bovine liver, 1577c) was used to validate elemental recovery and to correct for any batch variation.

### 2.5 Statistical analysis

All tests were performed using SPSS version 26 and GraphPad Prism version 8. Summary data are presented as means ± standard deviation (SD) or median and interquartile range (IQR) as appropriate. The Student's *t-*test or Mann-Whitney U-tests were applied depending on whether the data distribution was normal or skewed, as indicated by the Kolmogorov-Smirnov test. Spearman's Rank correlations tests were used to establish associations between continuous variables. The null hypothesis was rejected where P < 0.05.

## 3 Results

Baseline demographic and pregnancy outcome data are presented in Table 1.

Further detailed data regarding the whole cohort have been previously published [24]. Overall, the groups were matched for maternal age, BMI, and parity. By definition, women who had PE had significantly higher blood pressures (*P* < 0.05) and significant proteinuria. In addition, birth weights were also lower in the PE diagnostic group.

### 3.1 Gene expression

**Fig. 1:**
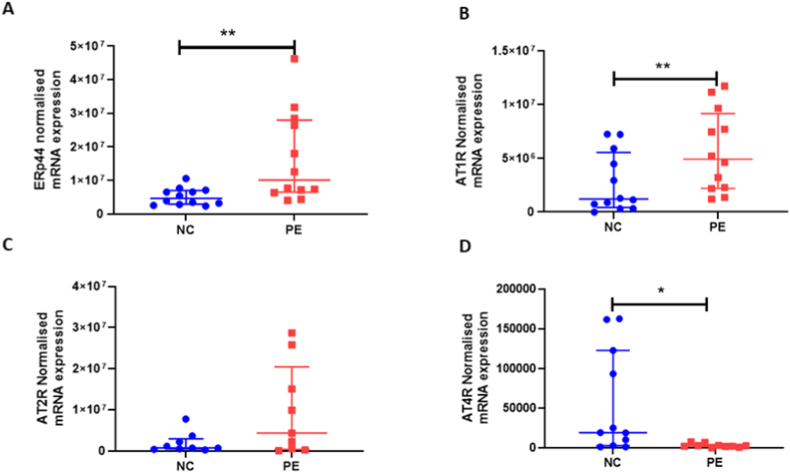
Placental mRNA expression of A) *ERp44*; B) *Angiotensin II type 1 receptor (AT1R)*; C) *Angiotensin II type 2 receptor (AT2R)* and D) *Angiotensin II type 4 receptor (AT4R).* Data presented as median [IQR] normalized copy number; *P < 0.05; **P < 0.001.

*ERp44* expression was higher in placentae from women with PE (median [IQR] normalized copy number; 10.1 × 10^6^ [6.5 × 10^6^, 28 × 10^6^]) compared to normotensive controls (4.7 × 10^6^ [2.9 × 10^6^, 7.0 × 10^6^]; P = 0.004. Fig. 1A). When the pre-eclampsia group was subdivided by early- (diagnosis <34 weeks; n = 6)/late-onset (diagnosis >34 weeks; n = 6) PE, the increased *ERp4*4 mRNA expression was only found in the late-onset (median [IQR]: 1.95 × 10^7^ [7.13 × 10^6^, 2.85 × 10^7^]) PE samples, compared to the normotensive controls (4.67 × 10^7^ [3.02 × 10^6^, 6.83 × 10^7^]; P = 0.007), and not early-onset PE (7.50 × 10^6^ [6.37 × 10^6^, 2.84 × 10^7^]; P > 0.05). Similarly, *AT1R* expression was increased in placenta from women with PE (5.2 × 10^6^ [2.3 × 10^6^, 7.7 × 10^6^]) compared to normotensive controls (2.9 × 10^6^ [8.8 × 10^5^, 5.9 × 10^6^]; *P* = 0.017; Fig. 1B). No differences were observed between groups for *AT2R* (*P* > 0.05; Fig. 1C). Conversely, *AT4R* expression was decreased in placentae from women with PE (2.5 × 10^3^ [1.9 × 10^3^, 3.2 × 10^3^]), compared to normotensive controls (1.9 × 10^4^ [6.6 × 10^3^, 1.1 × 10^5^]; *P* = 0.01; Fig. 1D).

A positive association between *ERp44* and *AT2R* expression (r = 0.612; *P* = 0.007) was observed. *AT1R* expression was negatively correlated with *AT4R* expression (r = −0.474; *P* = 0.03).

### 3.2 *ERp44* protein expression

**Fig. 2:**
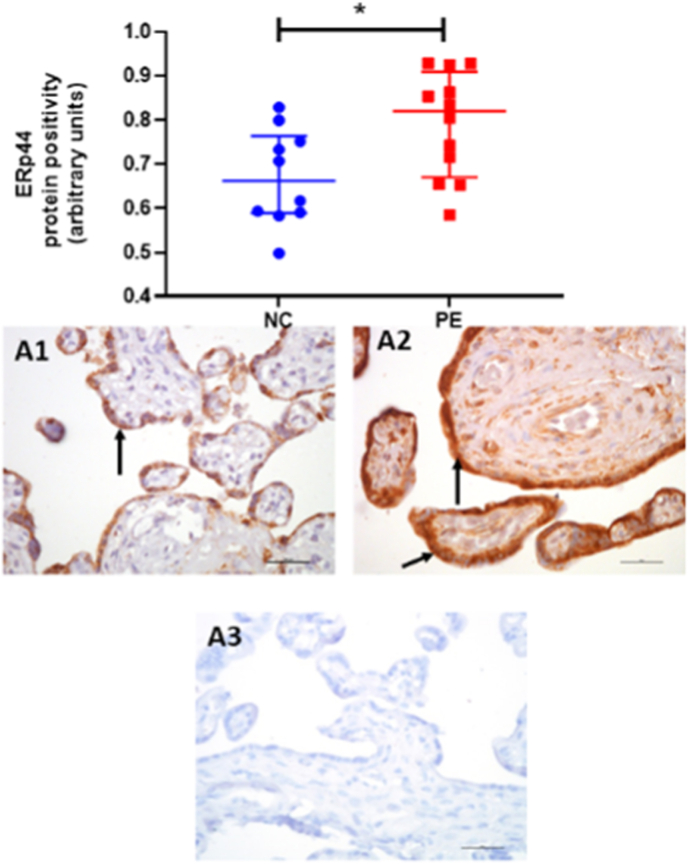
Placental protein expression and localization pf *ERp44* assessed by immunohistochemistry of *ERp44* in A1) normotensive controls (NC; n = 12); A2) women who had pre-eclampsia (PE; n = 12) and A3) IgG negative control. Data are presented as median [IQR]; *P < 0.05. Positive protein expression appears brown and is localised mainly to the syncytiotrophoblast (black arrow); scale bar = 100 μm.

Placental *ERp44* protein expression was confirmed in placental tissue, with strong immunohistochemical staining localised to the syncytiotrophoblast (Fig. 2).

Like mRNA, protein expression was increased in placentae from women with PE (median [IQR] positivity: 0.82 [0.69, 0.89]) when compared to normotensive controls (0.66 [0.59, 0.75]; *P* = 0.025; Fig. 2). When the pre-eclampsia group was subdivided by early-/late-onset PE (n = 6 for both), the increased ERp44 protein expression was found in both early- (0.86 [0.72, 0.93]; *P* = 0.22) and late-onset (0.79 [0.74, 0.84]; *P* = 0.042) PE samples, compared to the normotensive controls.

**Fig. 3:**
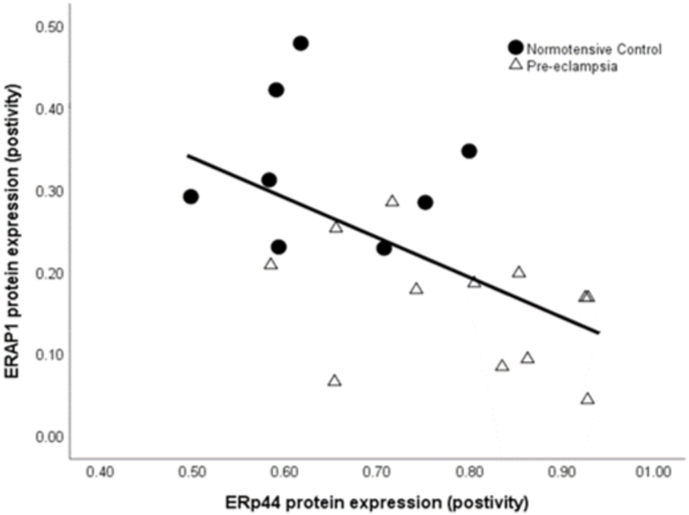
Scatter plot illustrations showing a negative correlation between *ERp44* and *ERAP1* protein expression (r = −0.61; *P* = 0.002). • Normotensive controls; Δ pre-eclampsia.

*ERp44* was found to be negatively correlated with previously measured *ERAP1* [7] placental protein expression (r = −0.61; *P* = 0.002; Fig. 3).

### 3.3 Placental zinc concentrations

**Fig. 4:**
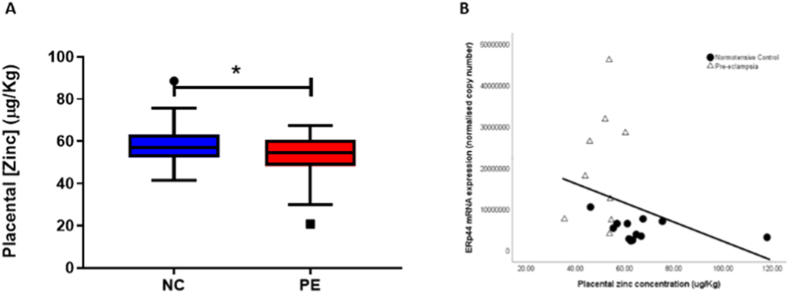
A) Zinc concentrations in placentae from normotensive controls (NC; n = 12) and women with pre-eclampsia (PE; n = 12). Data presented as median [IQR]; *P<0.05. B) Scatter plot illustrations showing a negative correlation between placental zinc concentrations and *ERp4*4 mRNA expression (r = - 0.63; *P* = 0.002). • Normotensive controls; Δ pre-eclampsia.

Zinc concentrations were lower in placentae from women with PE (median [IQR]: 53.7 [45.9, 54.2] μg/Kg), compared to normotensive women (62.9 [59, 67.1]; *P* = 0.001; Fig. 4A). When the pre-eclampsia group was subdivided by early-/late-onset PE (n = 6 for both), the decreased placental zinc concentrations were found in both early- (48 [38.8, 53.4]; *P* = 0.004) and late-onset (53.9 [53.7, 54.2]; *P* = 0.009) PE samples, compared to the normotensive controls.

In addition, a negative association was found between placental zinc concentrations and *ERp4*4 mRNA expression (r = - 0.627; *P* = 0.002; Fig. 4B).

## 4 Discussion

These novel data report that women who suffer from PE display higher ERp44 placental expression and lower placental zinc concentrations when compared to their normotensive controls. Additional, potentially important, associations with the RAS and *ERAP1* were also observed. We speculate these data suggest that dysregulation of placental expression of ERp44 contributes to the hypertension which is characteristic of PE. Our data supports previous reports of higher *ERp4*4 mRNA expression in placentae from women with PE [30,31]. The full function of *ERp44* in PE is still unclear, but increased *ERp44* in the placentae of women with PE may suggest that the trophoblasts need the *ERp44* activity as previously hypothesized [31]. *ERp44* can be induced by agents that cause the accumulation of unfolded proteins in the ER upregulating ER-resistance proteins during ER stress [32], which is a characteristic pathological finding of PE [33].

The negative association found between *ERp44* and *ERAP1* indicates that higher levels of *ERp44* correlate to decreased *ERAP1* expression. *ERp44* is a binding partner of *ERAP1* through disulfide bonds in the endoplasmic reticulum. It also controls the intracellular and extracellular localization of *ERAP1* [5] In the early secretory pathway of *ERp44*, zinc ions regulate the ability of *ERp44* to localize *ERAP1*, all of which occurs in the trophoblast cells of the placenta [13]. Our data suggest that even though *ERp44* is increased, the fact that zinc levels are decreased results in diminished *ERAP1* expression in PE patients. These observations may additionally contribute to the pathophysiological mechanism underlying hypertension. Future work is required to establish whether ERp44 and ERAP1 are co-localised and to determine if zinc is bound to ERp44 in the different groups.

The differences in the placental expression of RAS components in PE, confirm our previous data in a different sample cohort [8,12]. A negative correlation between *AT1R* and *AT4R* indicates an imbalance towards vasoconstriction in PE. Moreover, when *ERp44* complexes with the *ERAP1* protein, as noted in previous studies [7], it may result in less protein available to complete the conversion of Ang II. Hisatsune et al. have reported, in a sepsis study carried out in mice, that in *ERp44*^*+/−*^ mice (which express half the amount of *ERp44*) a much greater drop in blood pressure was observed compared to the *ERp44*^*+/+*^ mice with sepsis [7]. Thus, in PE, where there is systemic inflammation, the *ERp44*-*ERAP1* association is strengthened leading to lower availability of *ERAP1*. This *ERp44-ERAP1* complex contributes to reduced generation of Ang IV. Moreover, since AT4R is unique in that it is not a GPCR, but an insulin-regulated aminopeptidase, the AT4R that is secreted into the maternal circulation may also cleave AngIII to AngIV [34,35]. Thus with the reduced *AT4R,* this may provide less potential to counterbalance the activity of vasoconstrictive Ang II, raising blood pressure [5].

**Fig. 5:**
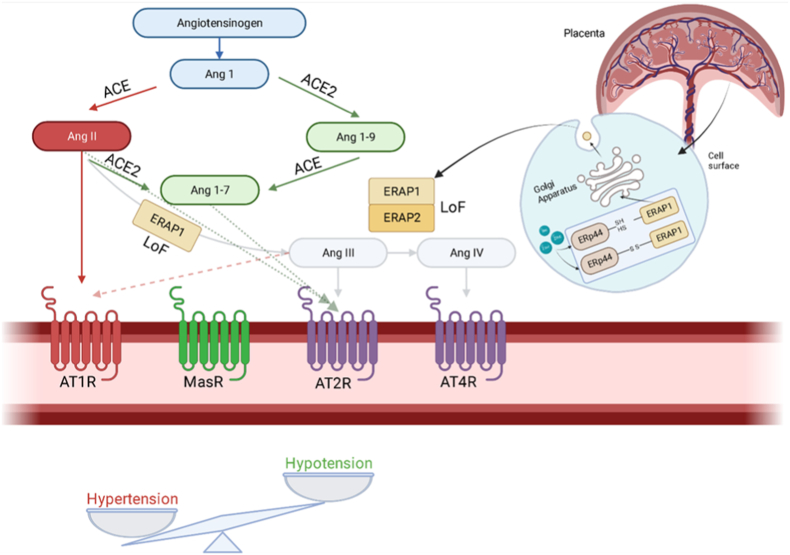
A summary diagram of the Renin-Angiotensin System (RAS) pathway and the *ERp44*/*ERAP1* complex illustrating how changes in zinc, can influence the *ERp44*-*ERAP1* complex, results in disruption in components of the RAS and blood pressure regulation. Angiotensinogen is converted into angiotensin I (Ang 1), through renin, which is then converted to the hypertension-promoting angiotensin II (Ang II) by angiotensin converting enzyme (ACE) or into Angiotensin 1-9 (Ang 1–9) through angiotensin converting enzyme 2 (ACE2), which promotes hypotensive effects. Both Ang II and Ang 1–9 convert to angiotensin 1-7 (Ang 1–7), which advances hypotension, through ACE2 and ACE, respectively. *ERAP1* is required to modulate the conversion of Ang II to angiotensin III (Ang III) and angiotensin IV (Ang IV). Ang III and Ang IV activate the Ang II type 1 and type 4 receptors (*AT2R* and *AT4R*), respectively, resulting in vasodilation. Due the negative association with *ERp44* and *ERAP1*, the higher levels of *ERp44* implicate lower levels of *ERAP1*, and this loss of function prevents Ang III and Ang IV from being formed thus disrupting the hypotensive effects. Instead, hypertension is exacerbated through Ang II, which activates *AT1R* and leads to increased blood pressure. Thus, we suggest that hypertension in pre-eclampsia results via the RAS and impaired *ERAP1*. Red functions indicate hypertensive effects and green functions indicate hypotensive effects. Gray functions delineate a lack of presence; LOF - Loss of Function.

Zinc deficiency has increased over the last decade due to a trend towards a zinc-poor diet, based on processed foods and soy-based substitutes as well as food grown in zinc-poor soil [15]. In this study, the lower placental zinc concentrations from women who had PE concurs with the findings of a previous study [36], as well as several reports of lower maternal zinc concentrations in PE [ [[37], [38], [39]]]. Zinc binds with high affinity to *ERp44*, modulating its localization and ability to retrieve proteins such as *ERAP1* [13]. Therefore, the lower placental zinc concentration may also contribute to the dysfunction of the *ERp44*/*ERAP1* complex, further exacerbating the hypertension in PE. Fig. 5 illustrates a schematic summarizing our proposed mechanism.

Other factors that have been reported to affect placental zinc concentrations are the expression and function of the zinc transporters ZnT 5, 6, 7 and 10, all of which have been shown to be upregulated in the Golgi in conditions of zinc deficiency or ER stress [40]. This is consistent with the physiological stress encountered in PE. Future work is required to fully elucidate the mechanistic relationship between the transporter with zinc and *ERp44*.

In summary, the increased expression in *ERp44* combined with lower placental zinc concentrations prevents an adequate release of the *ERAP1* protein. This *ERAP1* deficiency decreases the conversion of Ang II to Ang IV, contributing to the hypertension observed in PE.

## Funding

This work was produced by HDM under the terms of a 10.13039/501100000274BHF Basic Science Intermediate Basic Science Fellowship (FS /15/32/31604), 10.13039/100014013UK Research and Innovation Grand Challenges Research Fund GROW Award scheme (grant number: MR/P027938/1), NIHR–Wellcome Partnership for Global Health Research Collaborative Award (reference 217123/Z/19/Z) and International Research Collaboration Award from the 10.13039/501100000837University of Nottingham (IRCA 17_18/Jul18/003). LOK received an International Collaboration Research Grant from the 10.13039/501100000837University of Nottingham (ICR July2018) to travel to Virginia during this study. EDL received the Centers for Clinical and Translational Research (CCTR) Endowment Fund research grant titled “Defining the molecular mechanism of ERAP2 in immunological pathology of preeclampsia” (Grant Code: 2-94475) from the 10.13039/100009238Virginia Commonwealth University.

## Declaration of competing interest

None.
