# Supplementary material for: The differential placental expression of ERp44 and pre-eclampsia; association with placental zinc, the ERAP1 and the renin-angiotensin-system
Source: Placenta. 2023 Mar 24;134:9–14. doi: 10.1016/j.placenta.2023.02.006 (PMC10682376; doi:10.1016/j.placenta.2023.02.006)
Supplement: Multimedia component 1 [file mmc1.docx]

**Supplementary Table 1:** Details of primers used.

| **Gene** | **Accession number** | **Primers** | **Length (bp)** |
| --- | --- | --- | --- |
| ***ERp44*** | NM_015051 | 5'-agcccagagatacaggataagc-3'  5'-gttgcctgatgtaatctgcca-3' | 122 |
| ***AT1R*** | NM_004835 | 5'-ggctattgttcacccaatgaagt-3'  5'-tgggactcataatggaaagcac-3' | 177 |
| ***AT2R*** | NM_000686 | 5'-tatggcctgtttgtcctcattg-3'  5'-ccattgggcatatttctcaggt-3' | 115 |
| ***AT4R*** | NM_005575 | 5'-agtgcaactggttacaggcag-3'  5'-accacgatgacaaaagcacag-3' | 89 |
| ***B2M*** | NM_004048.2 | 5'-cttatgcacgcttaactatcttaacaa-3'  5'-taggagggctggcaacttag-3' | 127 |
| ***YWHAZ*** | NM_001135702.1 | 5'-acttttggtacattgtggcttcaa-3'  5'-ccgccaggacaaaccagtat-3' | 94 |
| ***GAPDH*** | NM_002046.3 | 5'-ggaagcttgtcatcaatggaa-3'  5'-tggactccacgacgtactca-3' | 102 |
